# Oral Health Clearance Outcomes for Cardiovascular Surgery

**DOI:** 10.1016/j.mayocpiqo.2024.01.002

**Published:** 2024-02-15

**Authors:** Miao Xian Zhou, Christopher F. Viozzi, Ondřej Heneberk, Sarah K. Lee, Kyle W. Klarich, Thomas J. Salinas

**Affiliations:** a Department of Dental Specialties, Mayo Clinic, Rochester, MN; b Division of Oral and Maxillofacial Surgery, Mayo Clinic, Rochester, MN; c Department of Cardiovascular Medicine, Mayo Clinic, Rochester, MN; d Department of Dentistry, University Hospital, Hradec Kralove, Czech Republic

## Abstract

**Objective:**

To determine the risk of morbidity and mortality in patients receiving dental extractions before planned cardiovascular surgery (CVS) and examine factors that may affect the chance of oral health clearance.

**Patients and Methods:**

A retrospective medical record review was performed of patients who underwent dental screening before CVS from January 1, 2015, to December 31, 2021, at a major medical institution. A total of 496 patients met the inclusion criteria and were divided into 2 groups. Group 1 patients were cleared to advance to planned CVS (n=390). Group 2 patients were not cleared for surgery and subsequently underwent dental extractions before planned CVS (n=106).

**Results:**

Six patients (5.7%) experienced postoperative complications after dental extraction that resulted in an emergency room visit. No deaths occurred after dental extraction before CVS. However, 4 patients died within 30 days of CVS, 3 from Group 1 (0.77%) and 1 from Group 2 (0.94%). Dental extraction before planned CVS showed a borderline significant association with death based on unadjusted (*P*=.06) and age-adjusted analysis (*P*=.05). Patients who reported seeing a dentist routinely had a significantly higher chance of oral health clearance (*P* <.001). No differences were noted between the 2 groups with regard to age, sex, or 30-day hospital readmission rate.

**Conclusion:**

Patients who had dental extractions completed before planned CVS may be at an increased risk of mortality. Further studies are needed to examine this relationship. Emphasis should be on prioritization of routine dental visits before planned CVS.

Cardiovascular disease (CVD) is one of the leading causes of death globally. Individuals with CVD often have other associated chronic comorbid conditions that place them at an increased risk for death.^1^ In a study examining the safety of tooth extraction in elderly patients with CVD, hypertension was found as the most common comorbidity, followed by coronary heart disease and arrhythmia.^2^

Oral bacteria such as *Streptococcus mutans* and *Aggregatibacter actinomycetemcomitans* have been associated with bacteremia and may be etiologic factors for the development of vascular inflammation resulting in CVDs.^3^, ^4^, ^5^ In addition, *Porphyromonas gingivalis,* a bacteria with a significant role in periodontal disease, has been detected in atherosclerotic plaque and may play a role in CVD.^6^ Entry of bacteria from the oral cavity to the bloodstream may occur when a tooth decays to the pulp or nerve and the capillary vessels are exposed allowing bacteria to enter the bloodstream.^7^

The American Heart Association has stated that a careful preoperative dental evaluation is recommended before cardiac surgery whenever possible to decrease the incidence of late postoperative endocarditis.^8^ Infections occurring in the perioperative period can increase morbidity, mortality, and cost.^9^^,^^10^ It is estimated that the average annual direct and indirect cost of CVD in the United States was $407.3 billion from 2018 to 2019 with hospital inpatient stays accounting for the highest direct cost ($111.4 billion) during that period.^11^ As such, a patient with planned cardiovascular surgery (CVS) may be referred to a dental provider for oral health clearance (OHC) before treatment to help rule out the presence of odontogenic infections.

Odontogenic infection is defined as infection around the alveolus, jaws, or face from a source that is derived from a tooth or its supporting tooth structure.^12^ These infections may be localized or they may spread to other sites resulting in sinusitis, difficulty in breathing, cavernous sinus thrombosis, sepsis, brain abscess, endocarditis, and even death. The dissemination may be through fascial spaces or by hematogenous or lymphatic routes. If odontogenic infections are identified, patients may be required to undergo urgent extractions to reduce the risk of oral bacteremia.

Although current evidence suggests an association between poor oral health and CVD, it is unclear whether patients with CVD can safely undergo dental procedures to achieve improved oral health. A study concluded that the rate of vascular events significantly increased in the first 4 weeks after invasive dental treatment but the authors pointed out the short-lived adverse effects are likely outweighed by the long-term benefits of invasive dental treatment.^13^ A previous study at our institution examined morbidity and mortality of patients undergoing dental extraction before cardiovascular surgical procedures (n=205), and found that patients with planned dental extraction before cardiac operation were at risk for major adverse outcomes, including a 3% risk of death before cardiac operation and an 8% risk of major adverse outcome.^14^ However, a more recent systematic review examining available evidence on the potential effect of dental treatment before cardiac valve surgery or left ventricular assist device implantation concluded that it is unclear whether postoperative outcomes differ in patients receiving dental treatment before cardiac surgery compared with those who do not receive any dental care.^15^

Although the available literature suggests the need to screen and treat dental infections in patients undergoing cardiovascular surgery, the protocol is not well established, and the effect of dental intervention on patient outcomes is not well understood.^16^ The primary aim of this study was to conduct a retrospective chart analysis to determine the risk of morbidity and mortality in patients receiving dental extractions with planned CVS. A secondary aim of this study was to determine patient factors that may affect their chance of OHC.

## Patients and Methods

This study was approved by the Mayo Clinic institutional review board (No. 22-001639), and all patients had previously consented to use of their records for research purposes.

A search of the electronic medical records database of patients that presented for dental screening before planned CVS from January 1, 2015, to December 31, 2021, were evaluated. Clearance was defined as free from acute symptomatic infections such as an abscess. If teeth were deemed nonrestorable or showed a high degree of mobility, removal may be indicated. When an individual underwent multiple dental screenings during this period, only the first dental visit and CVS was included in the analysis. Patients were included in the review and divided into 2 groups. Group 1 patients underwent a dental screening, were cleared for treatment, and underwent CVS. Group 2 patients underwent a dental screening, were not cleared for treatment, and underwent dental extraction before planned CVS. Patients were excluded from the review if they underwent a dental screening, were not cleared for treatment, and information is missing on whether dental extraction (if completed elsewhere) was performed before or after CVS or underwent a dental screening and were cleared to proceed but did not undergo CVS.

In this study, morbidity relates to postprocedural concerns defined as an emergent or urgent medical evaluation of symptoms that could logically accrue because of the oral surgical procedure and surgical morbidity, such as uncontrolled pain, bleeding, infection, or local wound issues (such as wound dehiscence) requiring reevaluation or retreatment.

All available records were reviewed by 2 reviewers (O.H. and M.X.Z.). The following information was recorded in the study database: sex, mortality date (if applicable), date of birth, date seen for dental screening examination, routine dental visits, date of last dental appointment, patient’s report on oral or tooth problem, CVS date, OHC status, date of extractions (if applicable), type of dental extraction setting (outpatient vs operating room), number of teeth or root tips removed, complications after dental extraction, 30-day hospital readmission after CVS, and New York Heart Association (NYHA) functional classification, when applicable.

### Statistical Analyses

Demographic characteristic summaries were provided for variables of interest which included mean (SD) and range for numeric values. For categorical values, totals and percentages were obtained and the amount of missing data. Logistic regression was performed to analyze associations between outcomes, including death after CVS, oral and tooth issues, and hospital readmission. Cox regression was performed to compare the time to discharge status between patients who were cleared (Group 1) versus those who were not cleared (Group 2) for CVS. All analyses were performed using R v 4.0.3 (R Foundation for Statistical Computing). Descriptive statistics and univariate and multivariate logistical regression analysis were conducted with significance set at *P*<.05.

## Results

Of the 496 individuals included after electronic health record review who presented to the Department of Dental Specialties, 390 patients were cleared for planned CVS (Group 1) and 106 patients were not cleared for planned CVS (Group 2) (Table 1). Group 1 had a mean (SD) age of 64.98 (18.28) years and Group 2 had a mean (SD) age of 64.56 (16.91) years. The mean ages in these 2 groups were not statistically significant (*P*=.83). No edentulous patients presented for OHC before planned CVS.

**Table 1:** Demographic Characteristics of Cardiovascular Patients Who Received and Did Not Receive OHC With Statistical Analysis of the 2 Groups^a^

Characteristic	Cleared, Group 1 (n=390)	Not cleared, Group 2 (n=106)	Total (N=496)	*P*
Age at CVS				
Mean ± SD	64.982±18.284	64.555±16.905	64.891±17.983	.83
Range	2.467-94.995	5.325-92.194	2.467-94.995	
Oral or tooth problem, n (%)				<.001^b^
No	311 (82.3)	61 (58.1)	372 (77.0)	
Yes	67 (17.7)	44 (41.9)	111 (23.0)	
Not reported, n	12	1	13	
Has a dentist they see routinely, n (%)				<.001^b^
No	91 (23.6)	48 (47.1)	139 (28.5)	
Yes	295 (76.4)	54 (52.9)	349 (71.5)	
Not reported, n	4	4	8	
Sex, n (%)				.93
Female	138 (35.4)	37 (34.9)	175 (35.3)	
Male	252 (64.6)	69 (65.1)	321 (64.7)	
Time since last dental appointment, n (%)				<.001^b^
0-6 mo	142 (41.4)	33 (34.4)	175 (39.9)	
>6-12 mo	27 (7.9)	3 (3.1)	30 (6.8)	
>12-23 mo	94 (27.4)	14 (14.6)	108 (24.6)	
24 mo—<5 y	55 (16.0)	20 (20.8)	75 (17.1)	
≥5 y	25 (7.3)	26 (27.1)	51 (11.6)	
Not reported, n	47	10	57	
Mortality up to 6 years after CVS, n (%)				.06
No	338 (86.7)	84 (79.2)	422 (85.1)	
Yes	52 (13.3)	22 (20.8)	74 (14.9)	
Mortality up to 1year after CVS, n (%)				.32
No	29 (55.8)	15 (68.2)	44 (59.5)	
Yes	23 (44.2)	7 (31.85)	30 (40.5)	
Not reported, n	338	84	422	
NYHA functional class, n (%)				.47
I	11 (12.6)	1 (4.3)	1 (4.3)	
I-II	1 (1.1)	0 (0.0)	1 (0.9)	
II	20 (23.0)	3 (13.0)	23 (20.9)	
II-III	2 (2.3)	0 (0.0)	2 (1.8)	
III	46 (52.9)	17 (73.9)	63 (57.3)	
III-IV	4 (4.6)	2 (8.7)	6 (5.5)	
IV	3 (3.4)	0 (0.0)	3 (2.7)	
Not reported, n	303	83	386	
Time from OHC examination to extraction				
Mean ± SD	NA	6.000±7.731		
Range	NA	0.000-37.000		
Not reported, n	390	3		
Time from CVS to death (in days)				.92
Mean ± SD	622.519±605.376	637.045±474.757	626.838±566.496	
Range	5.000-2311.000	8.000-1672.000	5.000-2311.000	
Not reported, n	338	84	422	

a Abbreviations: CVS, cardiovascular surgery; NA, not available; NYHA, New York Heart Association; OHC, oral health clearance.

b Significance at *P*<.05.

Group 2 underwent dental extractions with oral and maxillofacial surgery before planned CVS (Table 1). Patients waited an average of 6 days after OHC to undergo recommended dental extraction before CVS surgery, with a range of 0-37 days.

At *P*<.05, significant differences were found between the 2 groups with respect to 3 variables: (1) whether the patient self-reported oral or tooth problem, (2) whether the patient has a general dentist they visit routinely, and (3) time since their last dental visit. Group 1 showed 17.7% (69) reporting oral or tooth problem when compared with Group 2 at 41.9% (44) (*P*<.001). In Group 1 patients, 76.4% (298) reported having a dentist they saw routinely, whereas in Group 2, only 52.9% (56) patients reported this response (*P*<.001).

No deaths occurred after dental extraction before CVS. However, 4 patients died within 30 days of CVS, 3 from Group 1 (0.77%) and 1 from Group 2 (0.94%). Univariate regression was conducted at a *P* value of .05 (Table 2) and only age at CVS was statistically significant for increased odds of death (*P*<.001). A history of dental extraction before CVS showed a borderline significant association with death based on unadjusted (*P*=.06) and age-adjusted analysis (*P*=0.05). The odds ratio for age at CVS was 1.03, corresponding to an increased odds of death of 1.03 times for each year increase in age up to 6 years after CVS.

**Table 2:** Univariate Regression on Death Outcome Between the 2 Groups^a^

Variable	OR	95% CI	*P*
Extraction	1.702	0.965-2.928	.06
Age at CVS (per 1 year increase)	1.030	1.013-1.048	<.001^b^
Sex, Male	1.081	0.647-1.844	.77
Yes, to reported oral or tooth problems	1.116	0.61-1.96	.71
Yes, to seeing a dentist routinely	0.843	0.497-1.466	.54
Last dental appointment 6-12 months	1.433	0.493-3.662	.48
Last dental appointment >12-23 months	1.071	0.543-2.067	.84
Last dental appointment 24 month-<5 year	0.498	0.179-1.192	.14
Last dental appointment ≥5 year	1.763	0.796-3.75	.15
Time from dental examination to extraction (per 1 day)	1.049	0.992-1.11	.08
Time from extractions to CVS (per 1 day)	1	0.979-1.018	.98

a Abbreviations: CVS, cardiovascular surgery; OR, odds ratio.

b Significance at *P*<.05.

The number of patients with NYHA scores was noted in only 110 out of 496 patients, therefore further studies are needed to determine the importance of NYHA results on morbidity and mortality after dental extractions. In addition, using a univariate logistical regression, for self-reported oral or tooth problems, the odds ratio for OHC was 3.35 (95% CI, 2.09-5.35; P<.001) and 13 responses were missing.

A multivariate regression model was used to model the 2 items that displayed significance or borderline significance with univariate regression analysis (age at CVS and extraction status). In this model, for age at CVS, the odds ratio was 1.03 (95% CI, 1.01-1.05; P=.001). For extraction, the odds ratio was 1.78 (95% CI, 1.00-3.09; P=.05). In the extraction group, a mean of 5 teeth were extracted with 5 patients who had roots tips extracted (4 patients with 1 root tip and 1 patient with 2 root tips).

Six patients reported postextraction complications of pain, bleeding, swelling, and chest pain, resulting in follow-up visits to the emergency department or the provider care team (Table 3). Time (in days) from dental extraction to CVS was the following: mean (SD), 20.8 (25.1); median (IQR), 14.5 (5.25-25.0); and range, 1-180 days. Most extractions occurred in an outpatient setting with 91 listed as outpatient procedures and 15 listed as procedures performed in the operating room. The 30-day readmission rate between the 2 groups was not statistically significant (*P*=.40) (Table 4). A condensed table of the 3 significant findings noted between the 2 groups is found in Table 5 for the following: (1) self-reported problem; (2) routine dental care; and (3) time since their last dental visit. Group 2 had a longer time lapse since their last dental visit (≥2 years, 47.9% in Group 2 vs 23.3% in Group 1, *P*<.001).

**Table 3:** Postoperative Complications After Extraction Resulting in Emergent Visit (n=6)^a^^,^^b^

Individual/Sex	Complication and Action Taken
1/F	Bleeding: Seen in the emergency department 1 day after extraction of 5 teeth. The site was debrided, packings removed, and silver nitrate used and repacked with thrombin. The patient was on aspirin 81 mg once daily every evening.
2/M	Bleeding and pain: Seen in the emergency department 2 days after extraction of 8 teeth. The patient was on long-term anticoagulation–enoxaparin. Recommend an increase of acetaminophen 1000 mg every 6 hours and an increased dose of oxycodone 10 mg every 6 hours.
3/M	Chest pain: Seen in the emergency room later the same day after extraction. The patient reported on shortness of breath with chest pain after extraction of 32 teeth and alveoloplasty. A plan for stent placement and future myomectomy procedures was made. Pain was 0/10 at 1-month postoperative visit.
4/M	Bleeding: Seen in the emergency department 3 days after extraction of 10 teeth. The patient took warfarin and enoxaparin and was prescribed tranexamic acid.
5/M	Swelling: Seen in the emergency department 3 days after extraction of 7 teeth. The CT scan showed a possible hematoma which was confirmed clinically and resolved in 1 week. Prescribed a course of amoxicillin or clavulanate 875-125 mg twice daily for 3 days.
6/M	Pain: Seen in the emergency room later the same day after extraction of 4 teeth and was given an inferior alveolar nerve block using bupivacaine and epinephrine. The pain reduced from 8/10 to 2/10.

a Abbreviations: CT, computed tomography; F, female; M, male.

b Out of 106 individuals, or 5.7% of all individuals with extractions.

**Table 4:** Thirty-day Readmission Rate Between the 2 Groups^a^

	Group 1 (n=390), n (%)	Group 2 (n=106), n (%)	Total (N=496), n (%)	*P*
30-day readmission				.40
No	355 (91.7)	98 (94.2)	453 (92.3)	
Yes	32 (8.3)	6 (5.8)	38 (7.7)	
Missing data	3	2	5	

a Group 1 signifies the cleared group and Group 2 signifies the not-cleared group.

**Table 5:** Summary of the 3 Findings Between the 2 Groups on OHC (*P*<.001)

Finding	Cleared, Group 1 (n=390)	Not cleared, Group 2 (n=106)	Total (N=496)
Oral or tooth problem, n (%)			
No	311 (82.3)	61 (58.1)	372 (77.0)
Yes	67 (17.7)	44 (41.9)	111 (23.0)
Not reported, n	12	1	13
Has a dentist they see routinely, n (%)			
No	91 (23.6)	48 (47.1)	139 (28.5)
Yes	295 (76.4)	54 (52.9)	349 (71.5)
Not reported, n	4	4	8
Time since last dental appointment, n (%)			
0-6 mo	142 (41.4)	33 (34.4)	175 (39.9)
>6-12 mo	27 (7.9)	3 (3.1)	30 (6.8)
>12-23 mo	94 (27.4)	14 (14.6)	108 (24.6)
24 mo—<5 y	55 (16.0)	20 (20.8)	75 (17.1)
≥5 y	25 (7.3)	26 (27.1)	51 (11.6)
Not reported, n	47	10	57

Abbreviation: OHC, oral health clearance.

## Discussion

This study’s primary goal was to assess how dental extractions affect the risk of morbidity and mortality in CVS patients. Of the 496 individuals identified, 390 patients were dentally cleared to advance to CVS surgery and 106 patients were not cleared and underwent dental extractions before planned CVS. The percentage of patients reporting an oral or tooth problem, based on the patient’s subjective criteria, was 17.7%, a figure that was just slightly lower than the percent of patients that were not cleared in the study (21% or 106 of 496). This means that in a subset of patients, subjective sensation of an oral or tooth problem may not accurately translate to risk of oral disease. Patient assessment of oral concerns should prompt a visit to their general dentist. The definition of a dental need may be subjective on an individual basis. Pain associated with a tooth, or an asymptomatic chipped anterior tooth can be perceived as a dental need. However, in the context of OHC purposes for cardiovascular procedures, a dental-based infection that is within the tooth or the periodontium (soft tissue and surrounding alveolar bone) is the guiding baseline for a dental need. Therefore, routine visits to the patient’s general dentist are necessary to aid in a more objective assessment of dental needs that may put the patient at risk of oral infection.

In a previous study at our institution, 205 patients underwent 208 dental extractions before 206 planned cardiac operations with 12 patients dying within 30 days after dental extraction (6%).^14^ Out of these 12 patients, 6 (3%) occurred before cardiac operation and 3% occurred after cardiac operation.^14^ No control groups were noted in the previous study whereas our study compared mortality in patients who underwent dental extractions and those who did not undergo dental extractions. In our study, no deaths occurred after dental extraction before CVS. However, patients with a history of dental extraction before planned CVS showed a borderline significant association with death based on unadjusted (*P*=.06) and age-adjusted analysis (*P*=.05) when followed up to 6 years after CVS but no difference in mortality was noted between the two groups up to 1 year after CVS (*P*=.32).

Another study examining dental complications in patients who underwent surgical dental intervention from inpatient consults found that the elimination of oral infection before CVS did not appear to increase the risk of morbidity or mortality from time of dental consultation until 31 days post-CVS.^17^ In addition, a retrospective chart review found no significant difference in the number of medical adverse events or deaths experienced in patients who underwent dental treatment versus those who did not require dental treatment on patients with end-stage heart failure undergoing advanced surgical cardiac therapies.^18^

Dental procedures such as tooth extraction, scaling, periodontal surgery, endodontic therapy, rubber dam insertion, brushing, and chewing have all been found to result in bacteremia with the incidence of bacteremia after extraction ranging from 18% to 100%.^7^ In patients presenting with poor dental hygiene, periodontal or periapical infections may still cause bacteremia without having a dental procedure.^19^^,^^20^ In a study examining the role of oral hygiene in prevention of infective endocarditis, the presence of generalized bleeding after tooth brushing was associated with an almost 8-fold increased risk of developing bacteremia.^21^ A report in 2000 by the Surgeon General on Oral Health in America stated, “new research is pointing to associations between chronic oral infections and heart and lung disease, stroke, and low-birth weight, premature births.”^22^ Also stated in the report was that “efforts to improve cardiac care, for example, may include treatment of periodontal disease.” On the basis of a study examining the duration of bacteremia developing after dental intervention, a minimum 24-hour wait period is recommended before proceeding with medical or surgical intervention.^23^ In our study, the mean (SD) waiting period from the time of extraction to CVS was 20.8 (25.1) days, which may be because of scheduling conflicts and availabilities if patients needed to delay CVS until after dental extractions were completed to allow for healing after surgery.

Postoperative bleeding is a common complication after extraction in patients taking warfarin or anticoagulants. In the cohort of medically complex patients from our study, 3 cases out of the 106 (2.83%) patients who returned for follow-up visits to the emergency department or provider care team were managed for bleeding issues. The true cause of bleeding in any 1 patient is generally unknown; therefore, the cause is rarely noted in patient medical records. Postsurgical bleeding is always a result of some unclear interplay of patient factors (coagulation status vis a vis medications or other systemic factors, invasiveness of oral surgery performed, compliance with postoperative instructions such as diet and wound care). A recent study showed that there was no significant difference in postextraction bleeding in patient taking direct oral anticoagulants (ie, dabigatran, rivaroxaban, apixaban, and edoxaban) compared with those taking warfarin (1.9% vs 2.2%).^24^ Bleeding complications in anticoagulated or antiplatelet factor-treated patients after simple tooth extractions were found to be low.^25^ The frequency of hemorrhage after tooth extraction was similar in patients receiving continuous dabigatran or rivaroxaban compared with the warfarin group.^26^ A comparative study found that there is no need to alter the dosage of oral anticoagulants before dental extractions when the international normalized ratio is within the therapeutic range of 2.0-4.0 when done in a minimally traumatic manner with local measures for hemostasis to reduce risk of thromboembolic episodes in patients for patients taking warfarin.^27^ It has also been reported that dental extraction in patients that are thrombocytopenic is safe and that bleeding can be addressed with simple local measures.^28^

The secondary goal of this study was to identify patient factors that influenced the status of OHC. This study did show that the group without OHC had a longer lapse in time since their most recent dental visit (≥2 years, 47.9% vs 23.3%; *P*< 0.001), whereas more routine dental visits (defined as a dental visit during the previous 12 months) have been found to be associated with better oral health.^29^ Patients who reported poor oral health reported a 35% (hazard ratio, 1.35; 95% CI, 1.12-1.64) increase in the risk of CVD compared with individuals who self-reported excellent oral health.^30^ Another study found that in 147 patients diagnosed with CVD, 77.5% had periodontal disease and the presence of periodontal disease was also associated with a lower oral health-related quality of life.^31^ A nationwide population-based cohort study reported that after a median follow-up of 9.5 years, the risk of cardiovascular events was higher when a patient had periodontal disease, a higher number of dental caries, or more tooth loss.^32^ In the same study, regular dental visits, defined as once a year or more for professional cleaning, reduced cardiovascular risk by 14%.

A recent retrospective cohort study noted that participants who underwent dental screening had a lower incidence and risk of a major adverse cardiovascular event when compared with individuals who either did not undergo screening or underwent general health screening without dental evaluation during an 11-year follow-up period.^33^ These major adverse cardiovascular events were death, stroke, and myocardial infarction. Furthermore, when evaluating the predictions of oral health indicators versus known CVD risk factors for stroke mortality, oral health status consisting of over 10 tooth extractions was an independent predictor for cerebral infarction in addition to age, high-density lipoprotein-cholesterol, high-sensitivity C-reactive protein, and diabetes.^34^ The profile of teeth extracted in this study was a mean of 5 teeth lower than the risk factor found in the previous study by Haheim et al.^34^

The NYHA functional classification, first introduced in 1921, is frequently used to categorize patients with heart disease based on their symptoms and limitations.^35^ An important question to ask is whether patient’s NYHA classification plays a larger role in death status rather than age at CVS or whether they underwent an extraction. Given limited data on this variable, no conclusions can be drawn. Having a full data set with NYHA classifications on future studies would be needed to investigate this further.

Some limitations to our study included multiple types of cardiac surgeries; therefore, it was not feasible to provide a direct comparison between this study with studies focused on a certain procedure. Moreover, due to the referral of most patients from external sources, morbidity, mortality, or hospital readmission data may be incomplete in the electronic health record. In addition, the brief period between dental extractions and CVS in some patients can make the correlation of incidences of morbidity and mortality to the dental procedure or CVS unclear.

## Conclusion

Although the morbidity of dental extractions was low, the findings of this retrospective study indicate that patients who had extractions completed with the intention of achieving OHC for cardiovascular procedures may be at an increased risk of mortality. Interpretation of these results is cautioned by limitations of this study that include a relatively small sample size with several clinical confounders, retrospective data that is disproportionate between groups, and significant variation of performance status among patients afflicted with cardiovascular disease. The odds of OHC were significantly higher for patients who reported seeing their dentist routinely and for those without a self-reported oral or tooth problem. Using patient questionnaires, practitioners may be able to discern the likelihood of whether a patient may need to undergo dental extractions before CVS to avoid unnecessary delays in care. Future studies should also examine heart failure risk, medication exposure variables, patient comorbidities, antibiotic use, and possible health care exposures to determine their contribution and effect on patient morbidity, mortality, and OHC. With emerging research on the connection between oral health status and CVD, treatment should be geared toward achieving optimal oral health and prioritization of routine visits to a dentist to reduce the need for dental extractions. Future studies are needed to examine the relationship between oral health and cardiovascular risk.

## Potential Competing Interests

The authors report no conflicts of interest.
